# Surveillance as a Core Intervention to Strengthen Malaria Control Programs in Moderate to High Transmission Settings

**DOI:** 10.4269/ajtmh.22-0181

**Published:** 2022-06-06

**Authors:** Alison Fountain, Yazoume Ye, Arantxa Roca-Feltrer, Alexander K. Rowe, Alioune Camara, Aissata Fofana, Balthazar Candrinho, Busiku Hamainza, Medoune Ndiop, Richard Steketee, Julie Thwing

**Affiliations:** ^1^ Malaria Branch, Centers for Disease Control and Prevention, Atlanta, Georgia;; ^2^ ICF, Rockville, Maryland;; ^3^ Malaria Consortium, London, United Kingdom;; ^4^ The Global Fund to Fight AIDS, Tuberculosis and Malaria, Geneva, Switzerland;; ^5^ National Malaria Control Program, Conakry, Guinea;; ^6^ Research Triangle International, Conakry, Guinea;; ^7^ National Malaria Control Program, Maputo, Mozambique;; ^8^ National Malaria Elimination Centre, Lusaka, Zambia;; ^9^ National Malaria Control Program, Dakar, Senegal;; ^10^ President’s Malaria Initiative, Washington, District of Columbia

## Abstract

New tools are needed for malaria control, and recent improvements in malaria surveillance have opened the possibility of transforming surveillance into a core intervention. Implementing this strategy can be challenging in moderate to high transmission settings. However, there is a wealth of practical experience among national malaria control programs and partners working to improve and use malaria surveillance data to guide programming. Granular and timely data are critical to understanding geographic heterogeneity, appropriately defining and targeting interventions packages, and enabling timely decision-making at the operational level. Resources to be targeted based on surveillance data include vector control, case management commodities, outbreak responses, quality improvement interventions, and human resources, including community health workers, as they contribute to a more refined granularity of the surveillance system. Effectively transforming malaria surveillance into a core intervention will require strong global and national leadership, empowerment of subnational and local leaders, collaboration among development partners, and global coordination. Ensuring that national health systems include community health work can contribute to a successful transformation. It will require a strong supply chain to ensure that all suspected cases can be diagnosed and data reporting tools including appropriate electronic devices to provide timely data. Regular data quality audits, decentralized implementation, supportive supervision, data-informed decision-making processes, and harnessing technology for data analysis and visualization are needed to improve the capacity for data-driven decision-making at all levels. Finally, resources must be available to respond programmatically to these decisions.

## BACKGROUND

The 2020 World Malaria Report lauded the successes of malaria control during the past 2 decades, with substantial increases in access to insecticide-treated nets (ITNs) and indoor residual spraying, rapid diagnosis at the point of care, and treatment with artemisinin-based combination therapy (ACT), which have all led to an estimated 1.5 billion cases averted, 7.6 million lives saved, and a 60% reduction in malaria mortality.^1^ However, these gains are threatened by insecticide and drug resistance, invasive vectors, human population growth, stagnant funding, and now the COVID-19 pandemic, which has resulted in excess malaria mortality reported in the 2021 World Malaria Report.^2^ These threats have highlighted the need to define a resilient strategy and deploy resources more efficiently, based on differential needs at the subnational level informed by data and evidence.

The need for information to target malaria control efforts has driven the recognition of “surveillance as a core intervention” (SACI). The WHO Global Technical Strategy for Malaria 2016–2030 established Pillar 3 as “transform malaria surveillance into a core intervention.”^3^ Element 2 of the *High Burden to High Impact: A Targeted Malaria Response* states: “We are moving away from a ‘one-size-fits-all’ approach to malaria. Through better analysis and the strategic use of quality data, countries can pinpoint where to deploy the most effective malaria control tools for maximum impact. They can also use data to optimize the way tools are delivered to those in need through all conduits of delivery, including improved primary health care.”^4^

Detailed operational guidance exists for the use of SACI in low to very low transmission settings, including recommendations for case investigation, reactive case detection, and focus investigation,^5^^,^^6^ and there is a wealth of experience in countries close to elimination or having been certified as eliminated in the past 5 years, including Sri Lanka and Kyrgyzstan in 2016, Paraguay and Uzbekistan in 2018, Algeria and Argentina in 2019, and El Salvador and China in 2021.^6^ Some of these key strategies, such as targeting resources to foci of transmission, can be translated to other settings. However, further guidance is necessary to fully operationalize the concept of “surveillance as a core intervention” in moderate and high transmission settings. Documenting current understanding and practices of SACI in moderate and high transmission settings (*Plasmodium falciparum* prevalence > 5%) is also important in guiding continued improvement and adaptation of malaria surveillance systems and their use, as countries build toward elimination. This article presents perspectives, experiences, and best practices from national malaria control programs and international partners regarding SACI in moderate and high malaria transmission settings.

## METHODS

We contacted a group of experts in malaria surveillance, monitoring, and evaluation, from national malaria control programs (NMCPs) that were early adopters of routine surveillance-driven decision making, international non-governmental organizations, and funding partners who have guided efforts toward surveillance as a core intervention; all of the organizations contacted, with the exception of one NMCP, sent a response. Representatives from the NMCPs of Guinea, Mozambique, Senegal, and Zambia participated in this effort. We used a Delphi method type of approach to understand what SACI means in their contexts and practices and to learn from their experiences about the guiding principles and practical strategies they recommend to transform surveillance into an intervention in moderate to high transmission settings. These experts provided oral or written responses to a list of questions (see text box) in the language in which they felt most comfortable (November 2021–February 2022). These responses were collated, and the respondents were all included as coauthors.

**Guiding Questions**

1. What does “transforming malaria surveillance into a core intervention” mean in a moderate and high transmission context?
2. What strategies have you used or seen used to transform malaria surveillance into a core intervention in moderate and high transmission settings?
3. What strategies would you recommend for programs to practically accomplish this recommendation?
4. What are some guiding principles you would advise?
5. Are you aware of any literature providing guidance and/or examples of “surveillance as an intervention” in moderate and high transmission settings?
6. Any other thoughts or suggestions that you would like to provide for consideration?

## FINDINGS

### Historical context—introducing the concept of SACI for malaria control.

Although SACI may be relevant to other disease control programs where surveillance and interventions are intertwined (e.g., polio elimination), it was specifically invoked for the work against malaria. Early on, “interventions for malaria” included tools targeting mosquitoes (e.g., larvicides or insecticides for house walls or mosquito nets) or parasites (e.g., drugs for treatment or prevention). Other “interventions” were programmatic features that are part of health systems, such as supply systems to procure and transport malaria commodities (e.g., ITNs, rapid diagnostic tests [RDTs], ACTs); communications to inform people about when, where, and how to use the tools; or monitoring and evaluation activities to support disease surveillance, track intervention coverage, and evaluate the impact of malaria control programs.^7^^,^^8^

Before 2010, diagnostics were often unavailable at the point of care (POC), and many patients with febrile illness, especially children, were treated presumptively with antimalarial drugs, as recommended under the Integrated Management of Childhood Illness strategy to avoid excess mortality from delays in parasitological diagnosis.^9^ Microscopy was reserved for higher levels of the healthcare system for patients with severe disease or to support surveys or malaria field studies. The introduction of POC malaria RDTs in the early 2000s, and their increasing availability, improved the ability to direct antimalarial treatments to those with confirmed infection. In addition, data generated from the RDTs could be used to quantify the incidence of symptomatic malaria infection, diagnosed both in health facilities and by community health workers (CHWs). This new capacity of the passive surveillance system and the increasing comfort with the quality of RDTs evolved over time, and the technical parameters (sensitivity, specificity, predictive value of the tests) and relevant use of these are now quite well understood.

Surveillance, monitoring, and evaluation have historically been part of the overall health system, managed as a support unit in the ministry of health, such as the integrated disease surveillance and response (IDSR) unit or the Health Management Information System (HMIS) unit. The planning, implementation, financing, and ownership of surveillance data were not connected to the specific disease control programs. Consequently, the malaria program had limited input into exactly what, when, where, and how malaria-relevant data were collected and processed. As a component of the underfunded health system, the information quality was inadequate and not valued by the malaria program, its key stakeholders, or funders. For decades, the resource base for surveillance systems remained low and underfunded.

Before quality POC tests were widely available, monitoring the malaria burden was frequently done through cross-sectional household surveys every several years. These surveys produce high-quality population-level estimates for parasite prevalence and intervention coverage not available from routine surveillance systems. However, because of the relatively long interval between the survey and results dissemination, the generally sparse sampling to obtain national-level estimates, and the infrequency of such surveys, there was no direct or timely link between survey data and intervention implementation.

The increasing availability of POC RDTs has sparked a revolution in malaria surveillance, enabling improved targeting of antimalarials and the ability to “Test, Treat, Track,” as recommended by WHO in 2012. (“Malaria-endemic countries should ensure that every suspected malaria case is tested, that every confirmed case is treated with a quality-assured antimalarial medicine, and that the disease is tracked through timely and accurate surveillance systems to guide policy and operational decisions.”^10^) With the increasing use of quality RDTs and the adoption of the District Health Information Software 2, version 2 (DHIS2) information platforms for national case-based surveillance, confirmed malaria cases have been systematically reported in many countries—including case confirmation by health facilities and CHWs. Health facilities can often report hospitalized malaria cases, severe malaria, and malaria-attributed deaths (with or without a specific definition). Innovations such as RDTs and electronic health information platforms have set the stage to allow for novel, enhanced data-driven actions in response to surveillance activities.

High-quality and timely surveillance systems throughout the country do not just appear at the end of elimination. The 2017 WHO Framework for Malaria Elimination highlighted that “every country, including those with a high burden of malaria, may consider malaria elimination as a goal and adjust interventions to accelerate progress towards elimination.”^11^ The framework highlights that part of the responsibility of programs is to anticipate the needs for the next steps in reducing transmission toward elimination. Because surveillance systems are embedded in the overall health system, the transitions in the surveillance systems need to be anticipated and built well in advance of needing them for each next step. In addition, documenting these transitions is important so that overall trends can be adequately interpreted as data quality improves over time.

A malaria surveillance system must consider two types of information in its simplest terms. The first is the measurement of the extent of transmission and disease—measuring infections, illness, severe disease, and death. The second is measuring intervention coverage that specifically reduces the infections (transmission) and the consequent disease (illness, severe disease, and death). This second measurement also includes cross-sectional surveys that measure intervention coverage, entomological monitoring, and monitoring threats to current interventions (e.g., surveillance for drug and insecticide resistance, entomological surveillance, and HRP-2/3 deletions). However, the focus of this article is on the former—specifically, through the collection of routine data on cases detected at health facilities and the community level.

### Transforming malaria surveillance into a core intervention.

Correct case management is the foundation of good surveillance. Healthcare providers need clear, unambiguous case definitions of a suspected malaria case, confirmed malaria, severe malaria, admitted malaria, and death due to malaria. Most challenging is the definition of a suspected malaria case (one requiring diagnostic testing); case management guidelines often feature a vague definition, leaving the decision to test to the clinical intuition of the healthcare provider. Providers must receive adequate training on malaria case management, including case definitions and correct performance of RDTs. Adequate numbers of RDTs must be procured and distributed to allow providers to test all suspected cases. This requires attention both to accurate needs estimations and timely distribution, including considering the needs at the community level. Although these seem like commodity procurement and distribution concerns, disruptions to these supply chains have immediate and profound effects on the performance of malaria surveillance systems.

Next, the reporting system must be adequate to record and report the selected indicators, starting with the source document—outpatient department registers, which should have designated columns to facilitate recording and subsequent aggregation of suspected malaria cases and febrile illness, as well as the test performed, result of the test, and treatment provided. Historically, health facilities reported using paper forms or simple spreadsheets; more recently, ministries of health have adopted the DHIS2 platform for reporting routine data. Where infrastructure (electricity, Internet access) allows, this greatly facilitates timely reporting. However, health facilities usually lack infrastructure for direct data entry. Health facility register entries are aggregated into paper reports (monthly summary forms), which are sent to the district-level data managers for entry into DHIS2. This data aggregation from the registers to the summary paper forms presents a major data quality challenge of reporting accuracy. Technology such as ScanForm,^12^ which allows health facility staff to take and upload photographs of the register with automatic conversion to database structure, has been successfully piloted subnationally, but it requires production and distribution of specialized registers and might not be a scalable approach for all countries. There are also examples of using automated RDT readers to directly report test results into a central database^13^^,^^14^ that can be linked to DHIS2; however, the scale-up of this costly technology has proven to be challenging.

Timing and frequency of reporting is another element that programs must define when transitioning their surveillance systems: whether monthly data will suffice for decision-making purposes or whether they are able to collect and act upon weekly surveillance data. Most countries report nationally notifiable diseases through the IDSR system, although these diseases tend to be rare (e.g., measles, acute flaccid paralysis). Some countries have integrated weekly malaria reporting into the IDSR system alongside monthly reporting, but the challenge remains to reconcile the weekly and monthly numbers. Whether through the IDSR or alternate systems, countries are increasingly adopting malaria rapid reporting systems, at both the health facility and community levels, facilitating “near real-time” reporting of malaria data, to dynamically assess trends and priority areas and target responses. To minimize inefficiencies in the program, the timing of reporting should be designed to align with decisions based on that data and the frequency at which responses should occur.

Integrating CHWs and their case reporting data into the reporting structure must not be overlooked. Currently, the inclusion of CHW data in national reporting is inconsistent and incomplete. Although simple mobile platforms may greatly facilitate this, in most programs, CHWs rely on paper-based recording and reporting, often making timely reporting challenging. In most cases in which CHW data are included, these data are combined with the data of the supervising health facility for reporting purposes. However, keeping individual CHW data separate greatly improves the granularity of reporting, and some countries have started configuring the DHSI2 platform to this end and initiated pilots. This effort is in its infancy and requires urgent attention as countries optimize their community surveillance platforms to better understand malaria trends at the community level. Creative integration of data streams, including data not only from CHWs, but also from the private sector, entomology, interventions, and commodities, among others, could lead to a more comprehensive understanding and more effective approaches for malaria programs.

Although case definitions, diagnostic tools, and reporting infrastructure are foundational for surveillance, transforming surveillance into a core intervention requires timely analysis, interpretation, dissemination, decision-making, political support, and resources to act on those decisions. Personnel from POC to the national level need to know how to access, analyze, and interpret data to guide their decisions and be empowered and have the necessary resources to take action. Surveillance data have historically been reported in annual reports or periodic malaria bulletins, but their use in decision-making is limited. Cultivating a culture of data use at all levels is critical to making surveillance an intervention—using surveillance data effectively to guide the implementation of malaria interventions. In conjunction with fostering a culture of data use is ensuring data quality, both through centralized data quality assurance and data reviews at all levels (in addition to data checks made possible through digital platforms).

Malaria transmission in endemic settings is geographically heterogenous. Depending on the transmission intensity in a given geographic area, optimizing intervention coverage may require differing approaches. Surveillance guides program implementation by using data to stratify areas and apply recommended packages of interventions to those areas.^3^ Surveillance can then be used to track progress so that programs can course correct or strengthen best practices. In addition, surveillance is a powerful advocacy tool for investments in malaria elimination. In contexts with highly varied malaria transmission (e.g., Zambia and Senegal, and increasingly in other countries), a stratified approach to malaria programming uses more granular (sometimes individual-level) data to help target different intervention packages in areas of low compared with moderate to high transmission. Where there are “hot spots”—catchment areas with higher transmission in lower transmission zones—interventions can be intensified on a much more local level.^6^^,^^15^

Overarching these factors is the structural and political support to allow both the strengthening of the surveillance system and the actions to be taken in response to the data. Much of the complexity in implementing SACI comes from the broader contexts relating to who (and at what level) is empowered to make decisions, and resource issues (knowledge and skills, commodities, vehicles/fuel, and remuneration and motivation of facility and health system staff).

Finally, the surveillance system must anticipate the next set of information that will be required as transmission decreases, and new types of actions or responses are required or become available. This shift necessitates an intimate knowledge of the current burden and the desired targets to make the required decisions and investments. Although at high and moderate transmission levels, the primary interest is to ensure high coverage of interventions and adequate personnel, commodities, testing, and treatment, as malaria transmission declines, the system needs to become more granular. The surveillance system needs to be able to identify foci of local transmission, and local staff need to have the resources and ability to determine what can be done to stop that local transmission and implement it.

### Key innovations for transforming malaria surveillance into a core intervention.

Two key innovations have been deployed by countries that are successfully using surveillance as an intervention. First, while basic training in the use of the reporting tools (usually DHIS2) is a required element of personnel capacity building in surveillance, it is insufficient for ensuring quality reporting and use. Most programs have instituted regular data reviews in which personnel at all levels gather to review and audit data, give feedback, and discuss responses. In addition to data review and validation by supervisors, personnel may gather monthly, quarterly, or biannually (depending on the level of the health system) to present their data and conduct peer review. These regular data-driven deliberations among key actors and stakeholders to improve data quality, understand trends, and respond to program needs are key in fostering a culture of data use at all levels. This regular data interrogation has been strengthened through improved data visualizations and dynamic dashboard developments that are now either embedded into or added to DHIS2, such as the WHO Malaria module. These are useful in improving the quality and timeliness of the data because they are tailored to guide programming and decision-making. These data-driven discussions at lower levels help identify outbreaks and foci of higher transmission and develop a strategic response.

Second, many countries successfully employing surveillance as an intervention have extensively deployed CHWs, with substantial increases in the proportion of cases identified in the community (e.g., Rwanda, Senegal, Zambia). In these countries, the national policy usually allows CHWs to diagnose and treat patients of all ages, rather than limiting them to treating children under 5 years of age, enabling more comprehensive diagnosis, treatment, and reporting of cases by CHWs. This, in turn, requires greater attention to the supply chain to minimize potential disruptions and poor case management at the community level. Enhanced supervision of the CHWs is essential to ensure both quality of care and data reporting. CHWs are often placed primarily in remote communities, so there is a need to increase the number of CHWs to “saturate” the population with sufficient CHWs. Finally, programs will need to give better consideration to retention in collaboration with Ministry of Health and partners, paying CHWs a salary or offering some compensation so that the system does not frequently lose competent CHWs to other jobs, necessitating training of new CHWs. Because hiring and maintaining a robust, competent cadre of CHWs often is not within the purview of national malaria programs, collaboration with the Ministry of Health and partners supporting community health systems is recommended. In programs in which CHWs diagnose and treat a substantial proportion of malaria cases at the community level, and their reporting is integrated into the routine information system, community case management becomes an example of surveillance as a core intervention, leading to more timely decision-making at the local level.

## DISCUSSION

Effective use of surveillance as a core intervention depends on coordinating many interdependent processes and on the engagement of stakeholders from across the Ministry of Health and at all levels of the health system, as well as international financial and technical partners. A focus on malaria data quality and access to data can be a useful entry point toward surveillance as an intervention, but an approach that addresses the broader health system context is necessary to ensure a sustainable and effective intervention. If a possible response to surveillance data indicating increasing cases is to conduct indoor residual spraying or top-up insecticide-treated net distribution or simply to ensure that facilities and CHWs are stocked with diagnostic and treatment commodities, then partners, trained personnel, logistics, and a solid stock management system (with a surplus) are needed for this kind of rapid response or redistribution. Failure to respond not only prevents the surveillance system from acting as an intervention but demotivates personnel, leading to poor ownership and accountability.

The engagement, training, and ongoing motivation of many cadres in the health system—CHWs, providers, district health teams, and regional malaria officers—who are not on the payroll of or under the authority of the national malaria control program are other key components and are crucial to the success of surveillance as a core intervention. In addition to ensuring appropriate skills and knowledge among staff at each of these levels, the wider issues of turnover, motivation, remuneration, workload, and decision-making authority, among others, are likely to influence the success of SACI. Getting political buy-in for these initiatives and a commitment to using data for decision-making and resource allocation is a key—and challenging—component.

**Ten Recommendations for Transforming Surveillance into a Core Intervention**

1. Create national operational surveillance guidelines, including the following:
  a. Clear case definitions
  b. Reporting frequency adapted for context and needs (monthly, weekly)
  c. Data collection tools disaggregating by age group (e.g., < 5 years, 5–10 years, 5–14 years)
2. Develop a national course on surveillance and monitoring. Training of personnel at regional, district, and POC on the surveillance guidelines^16^ may build on existing efforts (e.g., MEASURE Malaria, Field Epidemiology Training Program). Provide periodic refresher trainings as standard operating procedures evolve over time.
3. Ensure that source documents, such as registers, tally sheets, and aggregation forms, correspond to case definitions and metrics adopted and are available at the reporting points (i.e., health facilities and community levels as applicable), and ensure that these correspond to data to be entered into the national surveillance platform (DHIS2).
4. Perform comprehensive ongoing needs analysis for malaria commodities, taking into account case definitions, to ensure RDT and ACT availability at all service delivery points, including health facilities and in the community.
5. Use information and communication technology to facilitate reporting (may include mobile phones and tablets at POC).
6. Scale up the routine surveillance system to community level through recruiting CHWs to provide diagnostic and treatment services and report in a timely manner.
7. Develop data visualization approaches that facilitate decision-making (e.g., traffic light systems for stockouts and outbreak alerts). Develop a regular surveillance bulletin for broad dissemination of data, including interpretation of trends.
8. Institutionalize regular data audits and reviews to validate reported data and embed data quality assessment findings into DHIS2 to inform interpretation of malaria trends.
9. Decentralize implementation and oversight of the system to the provinces and districts to foster ownership. The central level should provide technical support, supervision, and mentorship.
10. Conduct periodic assessments of the malaria surveillance system performance to inform key strategic decisions across all levels

Cost remains a significant consideration. Most, if not all, malaria programs consider the cost of an intervention before planning its implementation. Much of the guidance provided for scaling up surveillance provides a general guide and key issues to consider but does not address the practical costs required to implement or guidance for costing. The hiring to fill needed community work and the training, supervision, supplying, and remuneration of health workers should be documented across the national health system from the provincial, district, and health facility catchment areas (or relevant geographies) as appropriate to the country. A common understanding is required between national government leadership (Ministry of Health and Ministry of Finance at a minimum), donors, and local implementers regarding the critical nature of this information system, its quality, and stability over time.

National malaria control program leaders in endemic countries have rich experience in the practical application of transforming malaria surveillance into a core intervention. We have attempted to distill their experiences and recommendations in the interest of sharing their hard-earned wisdom with countries just starting down this road.
